# The Role of Neurotransmitters in the Protection of *Caenorhabditis Elegans* for *Salmonella* Infection by *Lactobacillus*

**DOI:** 10.3389/fcimb.2020.554052

**Published:** 2020-09-29

**Authors:** Xiaozhen Liu, Leming Jiang, Linyan Li, Hai Yu, Shaoping Nie, Mingyong Xie, Joshua Gong

**Affiliations:** ^1^Engineering Research Center of Health Food Design & Nutrition Regulation, School of Chemical Engineering and Energy Technology, Dongguan University of Technology, Dongguan, China; ^2^Guelph Research and Development Center, Agriculture and Agri-Food Canada, Guelph, ON, Canada; ^3^State Key Laboratory of Food Science and Technology, Nanchang University, Nanchang, China; ^4^National R&D Center for Freshwater Fish Processing, Jiangxi Normal University, Nanchang, China

**Keywords:** neurotransmitters, *Caenorhabditis elegans*, *Salmonella* Typhimurium, *Lactobacillus*, olfactory behavior

## Abstract

Salmonellosis is a common foodborne disease. We previously reported the protection of *Caenorhabditis elegans* from *Salmonella* Typhimurium DT104 infection by *Lactobacillus zeae* LB1. However, the mechanism is not fully understood. *C. elegans* exhibits behavior plasticity when presented with diverse pathogenic or commensal bacteria. Whether it can exert approach avoidance to *S*. Typhimurium through altering its neurological activity remains to be determined. In the current study, both the wild type and mutants defective in serotonin or dopamine production of *C. elegans* were used to investigate olfactory preference of the nematode to *L. zeae* LB1, DT104, and *Escherichia coli* OP50 by choice assays, and its resistance to DT104 infection and the protection offered by *L. zeae* LB1 using a life-span assay. The expression of target genes in *C. elegans* was also examined by real-time quantitative PCR. Results showed that pre-exposure to *L. zeae* LB1 did not elicit aversive olfactory behavior of the nematode toward DT104. Both mutants *tph-1* and *cat-2* succumbed faster than the wild type when infected with DT104. While pre-exposure to *L. zeae* LB1 significantly increased the survival of both the wild type and mutant *tph-1*, it provided no protection to mutant *cat-2*. Supplementation of dopamine resulted in both the resistance of mutant *cat-2* to *S*. Typhimurium infection and the protection from *L. zeae* LB1 to the same mutant. Gene expression data also supported the observations in the life-span assay. These results suggest that both serotonin and dopamine play a positive role in the host defense of *C. elegans* to *S*. Typhimurium infection and that the *L. zeae* LB1 protection is not dependent on modifying olfactory preference of the nematode but mediated by dopamine that may have involved the regulation of p38-mitogen-activated protein kinase and insulin/insulin-like growth factor signaling pathways.

## Introduction

Salmonellosis is one of the most common foodborne diseases, and its control still remains a challenge worldwide (Wei et al., 2019). Probiotics have long been used to control *Salmonella enteritidis* infection (Chambers and Gong, 2011; Forkus et al., 2017; Gut et al., 2018; Lai and Huang, 2019). However, their molecular mechanisms, including the roles of neurotransmitters in the host defense to *S. enteritidis* infection and probiotic protection, are not fully understood. Host–pathogen interaction is a dynamic process that includes not only the relationship between the host and pathogen but also their persistence during pathogenesis. The host innate immune response represents the first line of defense against pathogens' attack.

*Caenorhabditis* elegans is a laboratory animal, a good model to study the host–microbe interactions, including elucidations of their molecular mechanisms (Kaletta and Hengartner, 2006). Studies from *C. elegans* and other diverse organisms have revealed that key innate immune signaling regulators are strongly conserved (Irazoqui et al., 2010; Visvikis et al., 2014; Cohen and Troemel, 2015). The highly conserved mitogen-activated protein kinase (MAPK) signaling pathway that activates the innate immune response to bacterial infection is particularly important for exploring the role of innate immunity in organismal stress resistance and the regulation of longevity (Ermolaeva and Schumacher, 2014). Apart from the immunologic strategy to resist bacterial infection, *C. elegans* can also utilize its nervous system to respond to diverse microbial cues and to engage in a protective behavioral avoidance response to environmental pathogens (Meisel and Kim, 2014). Zhang et al. (2005) reported that *C. elegans* modified its olfactory preferences after an exposure to pathogenic bacteria. The nematode could avoid the odor from a pathogen and increase its attraction to the odor from familiar non-pathogenic bacteria. The increase of serotonin in ADF chemosensory neurons was shown to promote such aversive learning. In addition to the serotonin system, the dopamine system plays a critical role in motor control, reward, and cognition in vertebrate central nervous system (Schultz, 2016). Dysfunction of the dopamine system is associated with various disorders, including Parkinson's disease, schizophrenia, Tourette's syndrome, attention deficit hyperactivity disorder, and addictions (Sanyal et al., 2004). The dopamine-modulated behaviors are subject to behavioral plasticity in *C. elegans*. Qin and Wheeler (2007) have revealed that dopamine mediates *C. elegans* exploratory behavior and that the wild-type (WT) worms are significantly more likely than dopamine defective mutants to learn to make conditioned responses linking reward to location. However, little is known about the approach avoidance of *C. elegans* to *Salmonella* Typhimurium through altering the neurobehavior that is mediated by neurotransmitters.

Dopamine signaling in *C. elegans* has been shown to negatively regulate the innate immune response through a D1-like dopamine receptor, DOP-4, by down-regulating the p38/PMK-1 MAPK pathway, thus making the nematode more susceptible to *Pseudomonas aeruginosa* infection (Cao and Aballay, 2016). Similarly, serotonin signaling in *C. elegans* suppressed its innate immune response and limited the rate of pathogen clearance of *Microbacterium nematophilum* through regulating G-protein signaling (Anderson et al., 2013). It has been reported that conditioning of *C. elegans* with *Lactobacillus acidophilus* NCFM enhanced the Gram-positive immune responses of the nematode by activating both the p38 MAPK and the β-catenin signaling pathways (Kim and Mylonakis, 2012). Ikeda et al. (2007) also found that feeding nematodes with bifidobacteria or lactobacilli not only prolonged the life span of worms but also improved their resistance to the infection of *S. enteritidis*. Recently, we identified several *Lactobacillus* isolates that were able to protect *C. elegans* from death caused by *S*. Typhimurium or *Escherichia coli* infection (Wang et al., 2011; Zhou et al., 2014). *Lactobacillus zeae* LB1 was one of the best potential probiotic bacteria. It provided the protection by inhibiting the gene expression of *E. coli* enterotoxins (Zhou et al., 2014) and also by regulating *C. elegans* cell signaling through the p38-MAPK and DAF/insulin/insulin-like growth factor (IGF) pathways to control the production of antimicrobial peptides and defensing molecules (Zhou et al., 2018). However, it is unknown if the olfactory preference and neurotransmitters such as serotonin and dopamine play a role in the protection offered by the *Lactobacillus* isolate. The current study therefore wanted to address the questions.

## Materials and Methods

### *Caenorhabditis elegans* and Bacterial Cultures

*Caenorhabditis elegans* N2 Bristol WT, mutant *cat-2* (*e1112*, defective in dopamine production), and mutant *tph-1* (*mg280*, defective in serotonin production) were obtained from *Caenorhabditis* Genetics Center (CGC, University of Minnesota, Minneapolis) and cultivated as described (Julia et al., 2007). Briefly, worm embryos were collected by bleaching and were grown at 22°C. Worms were cultivated at 22°C on nematode growth medium (NGM) agar seeded with *Escherichia coli* OP50 (Stiernagle, 1999).

*Escherichia coli* OP50 grown in Luria–Bertani broth at 37°C for 12 h with a cell density of 10^8^ colony-forming unit (CFU) per milliliter was used as food for the nematode. *Salmonella enterica* serovar Typhimurium DT104 strain SA970934 is a porcine multiantibiotic-resistant isolate (Poppe et al., 1998, 2002). It was cultured on tryptic soy broth (TSB) or tryptic soy agar at 37°C for 16 h. Following three washes with S basal buffer containing 5.85 g of NaCl, 1 g of K_2_HPO_4_, 6 g of KH_2_PO_4_, and 1 ml of cholesterol (5 mg/ml in ethanol) in 1 L of the solution, a concentration of 10^8^ CFU/ml of cell suspension of the pathogen was used in assays. *Lactobacillus zeae* LB1 was cultured in de Man Rogosa Sharpe (MRS) broth or agar at 37°C for 18 h in an anaerobic chamber (Coy Laboratory Products, Grass Lake, MI) with an atmosphere of 85% N_2_, 10% CO_2_, and 5% H_2_ (Wang et al., 2011). After three washes with S basal buffer, a concentration of 1 × 10^9^ CFU/ml of cell suspension of the isolate was used in assays.

### Free-Choice and Two-Choice Behavior Assays

**Figure 1:**
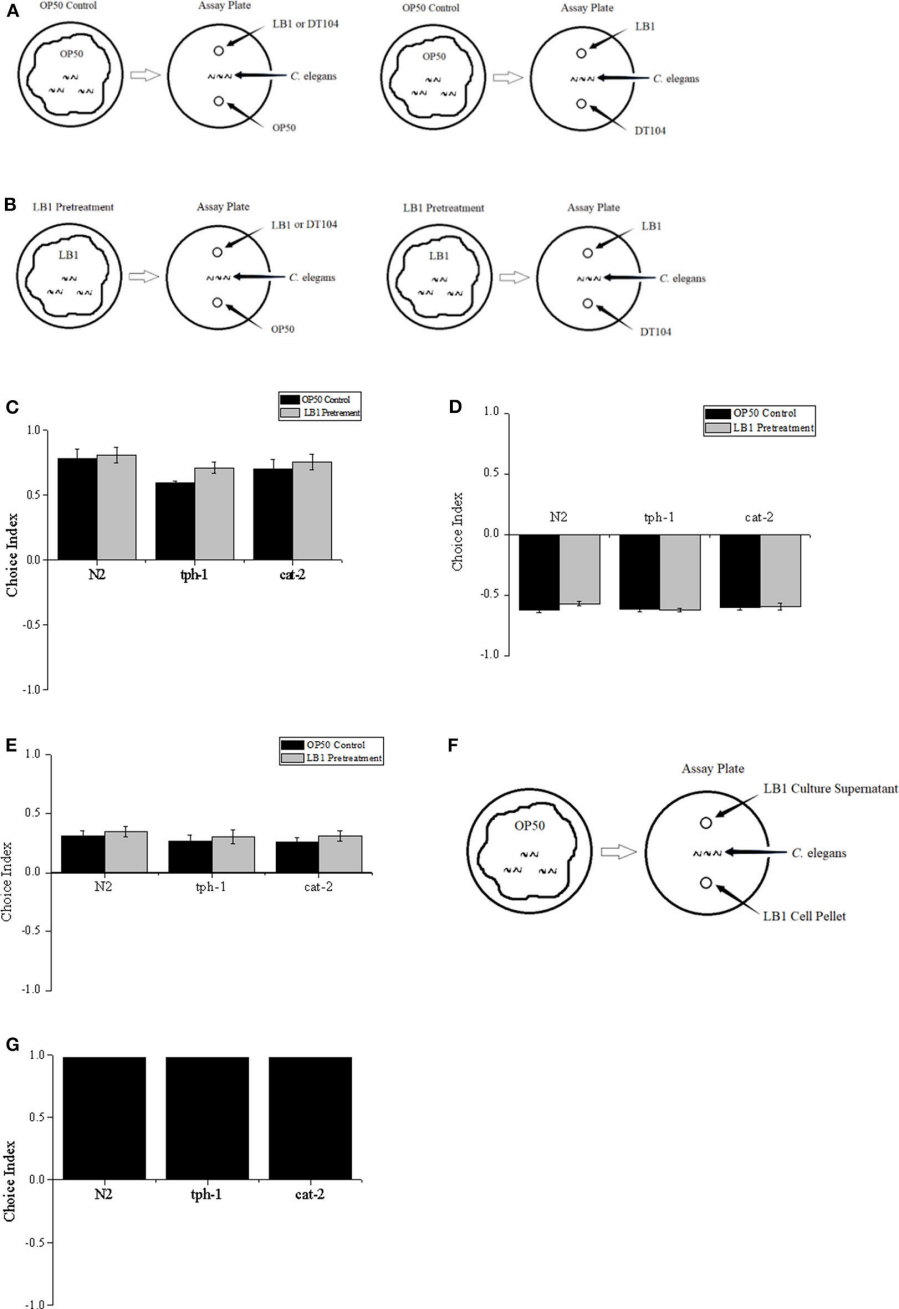
Two-choice behavior assay of *Caenorhabditis elegans*. L4 stage worms including the wild type (WT) (N2), mutant *tph-1*, and mutant *cat-2* were treated with *Escherichia coli* OP50 or *Lactobacillus zeae* LB1, and positioned in the middle of the assay plate, and exposed to test bacteria with equal distance; then choice index was calculated. **(A)** Worms were treated with OP50 only (OP50 control). **(B)** Worms were pretreated with *L. zeae* LB1. **(C)** Choice index of *L. zeae* LB1 against OP50. **(D)** Choice index of DT104 against *L. zeae* LB1. **(E)** Choice index of DT104 against OP50. **(F)** L4 stage worms were positioned in the middle of the plate and exposed to *L. zea*e LB1 cells or *L. zeae* LB1 culture supernatant with equal distance. **(G)** Choice index of *L. zeae* LB1 culture supernatant against *L. zeae* LB1 cells. Choice index of A against B = (number of worms in the lawn of bacterium A – number of worms in the lawn of bacterium B)/total worm number. Results are presented as mean ± S.D. with triplicate measurement.

L4 stage (Stiernagle, 1999) worms were grown on a NGM plate that was evenly spread with 200 μl of *E. coli* OP50 suspension. The free-choice or two-choice olfactory preference assay was based on standard chemotaxis assays (Bargmann et al., 1993) except that bacterial suspensions were used as odor sources. In the free-choice assay, 50 μl of each bacterial culture (*L. zeae* LB1, DT104, and OP50) was spotted onto one side of a new NGM plate and air-dried for 30 min at 25°C. Worms at L4 stage (50–100 worms) that had been washed twice in S basal buffer were then placed at the center of the plate, equidistant from the tested bacterial spots. The nematode was allowed to move freely for 1–2 h before being immobilized by 1 ml of 10 mM sodium azide applied at the bacterial spots. In most assays, worms quickly entered one bacterial lawn and remained there for the duration of the assay. A two-choice behavior assay was also conducted as indicated in Figures 1A–F. For pretreatment of *C. elegans*, 200 μl of *L. zeae* LB1 culture was spread on a plate, and the nematode was kept on the plate for 2 h at 25°C prior to the two-choice behavior assay. Each treatment was repeated three times. Choice index of A against B = (number of worms in the lawn of bacterium A – number of worms in the lawn of bacterium B)/total worm number. If choice index = −1.0 represents complete preference for bacterium B, then +1.0 represents complete preference for bacterium A, 0.0 represents an equal distribution (Julia et al., 2007; Kamaladevi et al., 2016).

### Life-Span Assay of *Caenorhabditis elegans*

The life-span assays of *C. elegans* were performed as described with slight modifications (Zhou et al., 2018). Synchronized *C. elegans* was transferred to NGM agar with *E. coli* OP50 at 25°C until it reached the L4 stage. In the assay, there were three types of treatment: (1) control, (2) DT104 infection only, and (3) *L. zeae* LB1 pretreatment. In the control, the nematode was treated with *E. coli* OP50 only throughout the entire assay. In the DT104 infection only, the L4 stage nematode was incubated with *E. coli* OP50 for 18 h followed by incubation with 200 μl of DT104 for up to 7 days in the absence of *E. coli* OP50. In the *L. zeae* LB1 pretreatment, the nematode was incubated with 200 μl of *L. zeae* LB1 for 18 h followed by incubation with 200 μl of DT104 for up to 7 days in the absence of *E. coli* OP50. The incubation temperature was 25°C. Each assay was started by transferring L4 stage worms (25 worms per replicate and three replicates per treatment) onto the agar plates seeded with either *E. coli* OP50 or *L. zeae* LB1, which was designated as day 0. After 18 h of incubation, worms on each plate within each treatment (either DT104 infection only or *L. zeae* LB1 pretreatment) were transferred to a fresh NGM plate daily that had been seeded with DT104 and was subsequently incubated at 25°C. The control group was processed in parallel, in which worms were transferred to a fresh NGM plate daily that had been seeded with *E. coli* OP50. The survival of nematode was examined at 24-h intervals for up to 7 days. To determine the survival of *C. elegans*, the number of live worms was recorded daily and calculated as follows: survival rate (%) = (live worms/total worms used) × 100. A worm was considered to be dead when it failed to respond to touch with a platinum loop; each treatment was repeated three times.

### RNA Extraction and Quantitative PCR Analysis

Fresh worms were washed three times with RNase-free water and lysed using the proteinase K-based method (Jiang et al., 2019) before RNA extraction with TRIzol™ Reagent (Invitrogen, Cat. # 15596026). After RNA extraction, the samples were treated with DNase I (Ambion, TX) at 37°C for 30 min and then verified as DNA-free by PCR assays. RNA integrity was determined by visualization in an agarose gel. The RNA concentration was determined with a NanoDrop ND-1000 spectrophotometer (NanoDrop Technologies, Wilmington, DE) after removing DNA contaminations.

Gene expression levels were determined by quantitative PCR (QPCR) analysis, following reverse transcription using qScript™ cDNA SuperMix (Quantabio, Cat. # 95048-100) according to manufacturer's protocol; 1–2 μl of cDNA template was used for a QPCR assay. Two housekeeping genes, gapdh and *rpl-4*, were used as references. QPCR assays were performed using a AB7500 Real Time PCR System (Applied Biosystems Inc., Foster City, CA) with iTaq™ Universal SYBR Green Supermix (Bio-Rad, Cat. # 172-5122) under the conditions as follows: 95°C for 3 min followed by 40 cycles of denaturing at 95°C for 30 s, annealing at 56°C for 1 min, and extension at 72°C for 30 s. The PCR primers are listed in Table 1.

**Table 1:** Primer of QPCR assay.

**Primer**	**Amplicon (bp)**	**Sequence (5^**′**^-3^**′**^)**	**Source (reference)**
Rpl-4-F	182	TTGCCCGTATTCCACGTGTT	This study
Rpl-4-R		GGATTCCGGAGGCAGCAATA	
Gapdh-F	158	ACTCGACCCACGGTCAATTC	This study
Gapdh-R		ACTCGACAACGAAATCGGCT	
Daf-16-F	181	TCGTCTCGTGTTTCTCCAGC	Zhou et al., 2018
Daf-16-R		TAATCGGCTTCGACTCCTGC	
Age-1-F	359	CTCCTGAACCGACTGCCAAT	Zhou et al., 2018
Age-1-R		AAATGCGAGTTCGGAGAGCA	
Lys-7-F	153	GTACAGCGGTGGAGTCACTG	Zhou et al., 2018
Lys-7-R		GCCTTGAGCACATTTCCAGC	
Clec-60-F	219	CGGTTTCAATGCGGTATGGC	Zhou et al., 2018
Clec-60-R		TGAAGCTGTGGTTGAGGCAT	
Clec-85-F	121	CCAATGGGATGACGGAACCA	Zhou et al., 2018
Clec-85-R		CTTCTGTCCAGCCAACGTCT	
Abf-3-F	189	AACAGATTGGGGTCAGCTCG	Zhou et al., 2018
Abf-3-R		TGGAGACCATTATTGCCGGG	
Abf-2-F	176	CCGTTCCCTTTTCCTTGCAC	Zhou et al., 2018
Abf-2-R		GACGACCGCTTCGTTTCTTG	
Tir-1-F	223	TTGGGTGCACAAAGAGCTGA	Zhou et al., 2018
Tir-1-R		GGTCGGTGTCGTTCTGTTCA	
Nsy-1-F	122	AGCGGCTCGATCAACAAGAA	Zhou et al., 2018
Nsy-1-R		CCCATTCCACCGATATGCGA	
Sek-1-F	158	CACTGTTTGGCGACGATGAG	Zhou et al., 2018
Sek-1-R		ATTCCGTCCACGTTGCTGAT	
Pmk-1-F	115	CCAAAAATGACTCGCCGTGA	Zhou et al., 2018
Pmk-1-R		CTTTTGCAGTTGGACGACGA	
Skn-1-F	153	CTGGCATCCTCTACCACCAC	Zhou et al., 2018
Skn-1-R		TTGGTGATGATGGCCGTGTT	
Dbl-1-F	194	TTTTGCGGCGAACAAATCGT	Zhou et al., 2018
Dbl-1-R		TTCGCTGTTGCCTGTTTGTG	
Spp-1-F	106	TGGACTATGCTGTTGCCGTT	Zhou et al., 2018
Spp-1-R		ACGCCTTGTCTGGAGAATCC	
Jnk-F	102	TCACAACACTCTGCTCGCAT	Zhou et al., 2018
Jnk-R		TGGAACCAGCCAATTCCCAA	
Sod-3-F	73	AAATGTCCGCCCAGACTATG	Zhou et al., 2018
Sod-3-R		TGGCAAATCTCTCGCTGA	
Ced-3-F	166	AGAAGGAGCTTGCTAGAGAGGA	Zhou et al., 2018
Ced-3-R		ACTGCTTTCACGATCTCCCG	

The fold changes of target genes were determined using the 2^−ΔΔCt^ method (Livak and Schmittgen, 2001). The ΔCt represents the difference between the Ct value with the primers to a target gene and the Ct value to the housekeeping genes. The ΔΔCt represents the difference between the ΔCt value of mutant *cat-2* group in either the presence or absence of dopamine and the ΔCt value of WT (N2) control group within the same treatment. The WT groups had the 2^−ΔΔCt^ value of 1.

### Functional Restoring Experiments

Previous studies have shown that exogenous dopamine added to experimental plates or buffers can mimic the effects of a dopaminergic signaling, although a relatively high concentration is required to elicit a response in the assay due to the low permeability of cuticle to dopamine (Sanyal et al., 2004; Ezak and Ferkey, 2010; Ezcurra et al., 2011). In the current study, dopamine hydrochloride (Sigma-Aldrich, CAS: 62-31-7) solution was sterilized through 0.22-μm membrane filters and included freshly in NGM agar at 0, 0.25, 0.5, 0.75, and 1.5 mM. Synchronized L1 stage worms of mutant *cat-2* were cultured on NGM agar containing dopamine and seeded with OP50 until they reached L4 stage. In the life-span assay, the entire procedure of the mutant, including *L. zeae* LB1 pretreatment, DT104 infection, and subsequent observation, was conducted in the presence of dopamine. The WT was treated in parallel without exposure to dopamine to serve as a reference.

### Statistical Analysis

All statistical computation analyses were performed using Statistical Product and Service Solutions (SPSS, Windows version 11.5). Survival curves of *C. elegans* were compared using the Kaplan–Meier survival analysis followed by a log-rank test. One-way analysis of variance and the Tukey multiple comparisons were carried out to test for significant differences between the means. Means with *P* ≤ 0.05 were considered to differ significantly, and *P* ≤ 0.01 were considered to be extremely different.

## Results

### Olfactory Preference of *Caenorhabditis elegans*

The preference of *Caenorhabditis elegans* to different bacterial odor was firstly investigated in a free-choice assay. *C. elegans* was significantly attracted to *Lactobacillus zeae* LB1 (*P* ≤ 0.05), but not to other bacteria. Approximately 92% worms moved to *L. zeae* LB1, 5% to DT104, and 3% to OP50 in the assay.

To determine whether pretreatment with *L. zeae* LB1 can alter the preference of *C. elegans* toward DT104, binary choice assays without (Figure 1A) or with (Figure 1B) *L. zeae* LB1 pretreatment were conducted. Although most worms were attracted to *L. zeae* LB1 in the assay, the *L. zeae* LB1 pretreatment did not change the olfactory behavior of *C. elegans*. The choice indexes in each pair of the test were similar regardless if the nematode had been treated with *L. zeae* LB1 or not, in which the choice indexes were 0.81 and 0.78 for the pair of *L. zeae* LB1 vs. OP 50 with or without *L. zeae* LB1 pretreatment (Figure 1C), −0.57 and −0.62 for the pair of DT104 vs. *L. zeae* LB1 with or without *L. zeae* LB1 pretreatment (Figure 1D), and 0.35 and 0.31 for the pair of DT104 vs. OP50 with or without *L. zeae* LB1 pretreatment (Figure 1E). The olfactory behavior of *C. elegans* mutants defective in serotonin (*tph-1*) or dopamine (*cat-2*) was also investigated in the two-choice assay. Both mutants exhibited similar choice behavior to the WT to the tested bacteria (Figures 1C–E) regardless of the *L. zeae* LB1 pretreatment.

To determine which part of the *L. zeae* LB1 culture attracted *C. elegans, L. zeae* LB1 cells and culture supernatant were tested individually (Figure 1F). The choice index of *L. zeae* LB1 culture supernatant against its cells was almost 1. Similar results were also obtained when both mutants (*tph-1* and *cat-2*) were examined in the same assay (Figure 1G).

### The Potential Involvement of Serotonin and Dopamine in the Resistance of *Caenorhabditis elegans* to *Salmonella* Typhimurium DT104 Infection

**Figure 2:**
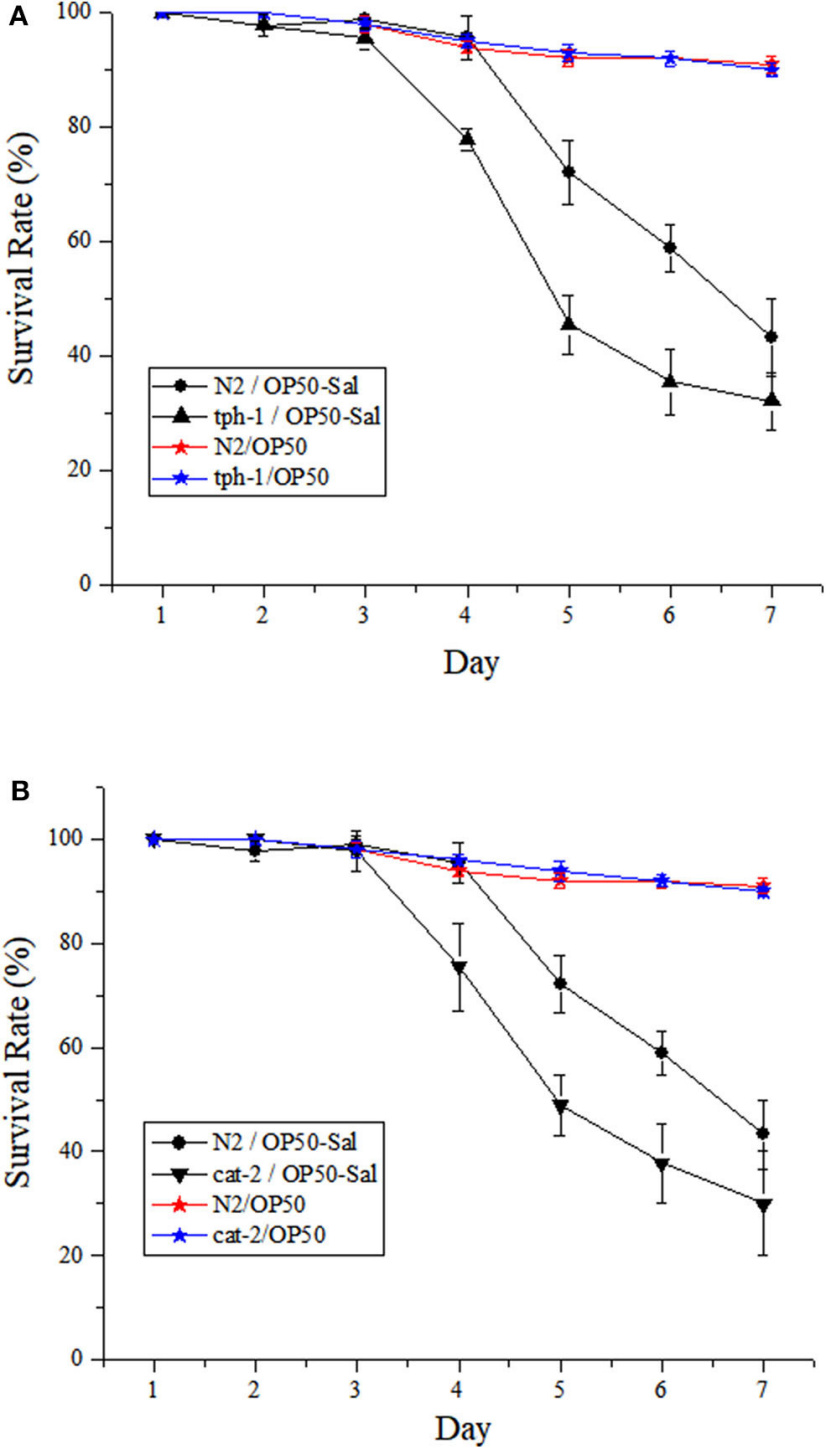
The potential involvement of serotonin and dopamine in the resistance of *Caenorhabditis elegans* to *Salmonella* Typhimurium DT104 infection. The mutants are defective in **(A)** *tph-1* or **(B)** *cat-2*. N2: the wild type (WT) of *C. elegans*. N2/OP50, *tph-1*/OP50, and *cat-2*/OP50: the WT or each mutant was treated with OP50 only. N2/OP50-Sal, *tph-1*/OP50-Sal, and *cat-2*/OP50-Sal: the WT or each mutant was incubated with OP50 first, followed by DT104 infection in the life-span assay. The number of live worms was recorded daily and calculated as follows: survival rate (%) = (live worms/total worms used) × 100. A worm was considered to be dead when it failed to respond to touch. Results are presented as mean ± S.D. with triplicate measurement.

The responses of both the WT and mutants of *C. elegans* to DT104 infection are shown in Figure 2. The WT infected with DT104 had a significantly shorter life span than uninfected worms (feed *Escherichia coli* OP50 only; *P* ≤ 0.05). For example, the survival rate of infected WT was 43.33 ± 6.67% at the seventh day, while that of uninfected WT was 91.00 ± 1.51%. Mutants *tph-1* and *cat-2* both succumbed faster than the WT after DT104 infection (*P* ≤ 0.05). At the seventh day, *tph-1* and *cat-2* had a survival rate of 32.22 ± 5.03% (Figure 2A) and 30.00 ± 6.00% (Figure 2B), respectively.

### The Potential Involvement of Serotonin and Dopamine in the Protection From *Lactobacillus zeae* B1

**Figure 3:**
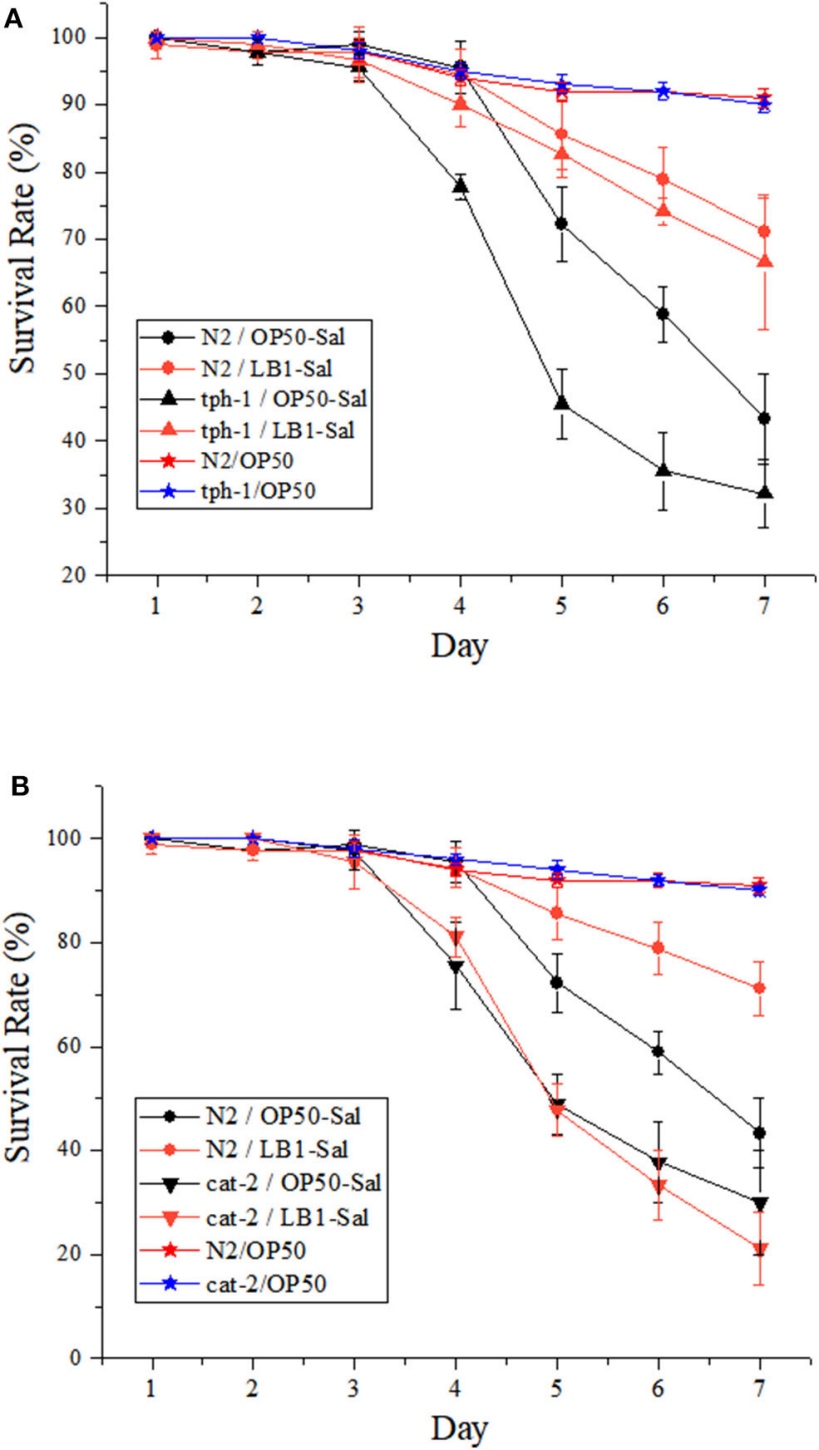
Effect of *Lactobacillus zeae* LB1 on the resistance of *Caenorhabditis elegans* mutants to *Salmonella* Typhimurium DT104 infection. The mutants are defective in **(A)** *tph-1* or **(B)** *cat-2*. N2: the wild type (WT) of *C. elegans*. N2/OP50, *tph-1*/OP50, and *cat-2*/OP50: the WT or each mutant was treated with OP50 only. N2/OP50-Sal, *tph-1*/OP50-Sal, and *cat-2*/OP50-Sal: the WT or each mutant was incubated with OP50 first, followed by DT104 infection in the life-span assay; N2/LB1-Sal, *tph-1*/LB1-Sal, and *cat-2*/LB1-Sal: the WT or each mutant was incubated with *L. zeae* LB1 first, followed by DT104 infection in the life-span assay. The number of live worms in each group was recorded daily and calculated as follows: survival rate (%) = (live worms/total worms used) × 100. A worm was considered to be dead when it failed to respond to touch. Results are presented as mean ± S.D. with triplicate measurement.

The effects of *L. zeae* LB1 on the resistance of *C. elegans* (both the WT and mutants) to DT104 infection were also investigated. Synchronized L4 stage WT worms were treated with *L. zeae* LB1 for 18 h followed by *Salmonella* Typhimurium DT104 infection for up to 7 days. The results showed that the survival rate of the WT pretreated with *L. zeae* LB1 was higher than that of the same worms infected with DT104 only, indicating that *L. zeae* LB1 pretreatment for 18 h significantly increased the resistance of the WT to *S*. Typhimurium infection (*P* ≤ 0.05) as reported previously (Wang et al., 2011). Pre-exposure to *L. zeae* LB1 for 18 h could significantly increase the survival of mutant *tph-1* with the level close to the WT, which was significantly higher than that of mutant *tph-1* infected with DT104 only (*P* ≤ 0.05; Figure 3A). In contrast, *L. zeae* LB1 offered no protection to mutant *cat-2*, resulting in a survival rate comparable with that of the same mutant infected with DT104 only (Figure 3B). These results suggested that dopamine, but not serotonin, was involved in the protection offered by *L. zeae* LB1 to *C. elegans* against *S*. Typhimurium DT104 infection.

### Supplementation of Dopamine Increased the Resistance of Mutant *cat-2* to *Salmonella* Typhimurium DT104 Infection

**Figure 4:**
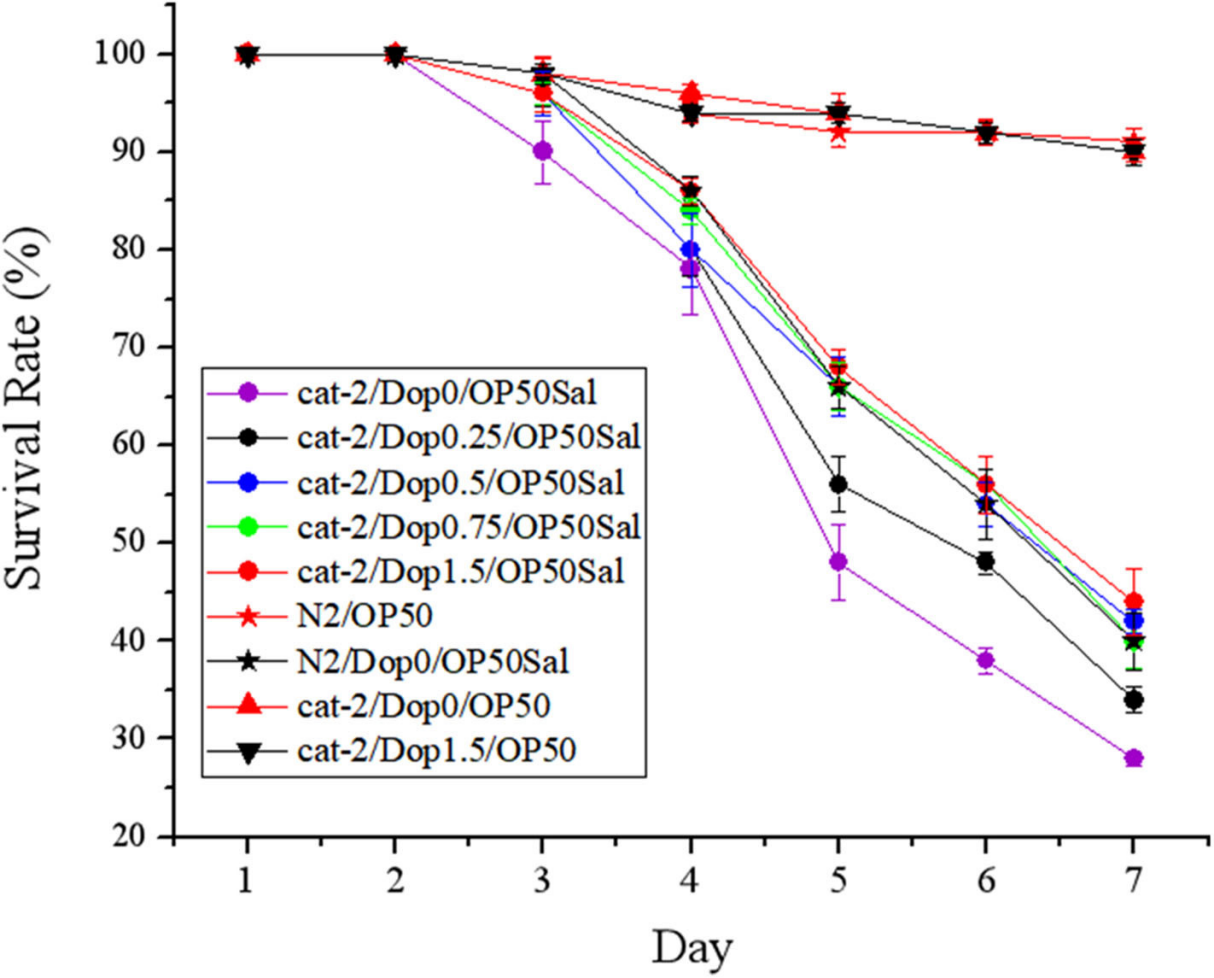
Dopamine involved in the resistance of mutant *cat-2* against *Salmonella* Typhimurium DT104 infection. Synchronized L1 stage worms of mutant *cat-2* were treated with various concentration of dopamine (0, 0.25, 0.5, 0.75, and 1.5 mM) in nematode growth medium (NGM) agar seeded with OP50 until they reached L4 stage. And then the worms were infected with DT104 for 7 days. Then life span was detected. N2: the wild type (WT); *cat-2*: mutant; Dop0: dopamine at 0 mM; Dop0.25: dopamine at 0.25 mM; Dop0.5: dopamine at 0.5 mM; Dop0.75: dopamine at 0.75 mM; Dop1.5: dopamine at 1.5 mM; OP50Sal: treated with *Escherichia coli* OP50 followed by DT104 infection; OP50: treated with *E. coli* OP50 only. Wild-type worms (N2) were used as control. The number of live worms in each group was recorded daily and calculated as follows: survival rate (%) = (live worms/total worms used) × 100. A worm was considered to be dead when it failed to respond to touch. Results are presented as mean ± S.D. with triplicate measurement.

To determine the involvement of dopamine in the host defense to *S*. Typhimurium DT104 infection, a functional restoring experiment was conducted by including different concentrations of dopamine (0, 0.25, 0.5, 0.75, and 1.5 mM) in the life-span assay of *C. elegans*. As shown in Figure 4, mutant *cat-2* defective in dopamine production succumbed faster than the WT to *S*. Typhimurium infection (*P* ≤ 0.05), which was in agreement with the observation shown in Figure 2B. The supplementation of dopamine at 0.25 mM significantly increased the resistance of *cat-2* to the infection (*P* ≤ 0.05), in which the survival rate was 34.00 ± 1.34% at the seventh day compared with 28.00 ± 0.68% in the group without dopamine supplementation. When the concentration of dopamine reached 0.5 mM and above (up to 1.5 mM), the resistance of *cat-2* was further enhanced (*P* ≤ 0.05) with the level similar to the WT infected with DT104 only.

### Supplementation of Dopamine Resulted in the Protection From *Lactobacillus zeae* LB1 to Mutant *cat-2* Against *Salmonella* Typhimurium DT104 Infection

**Figure 5:**
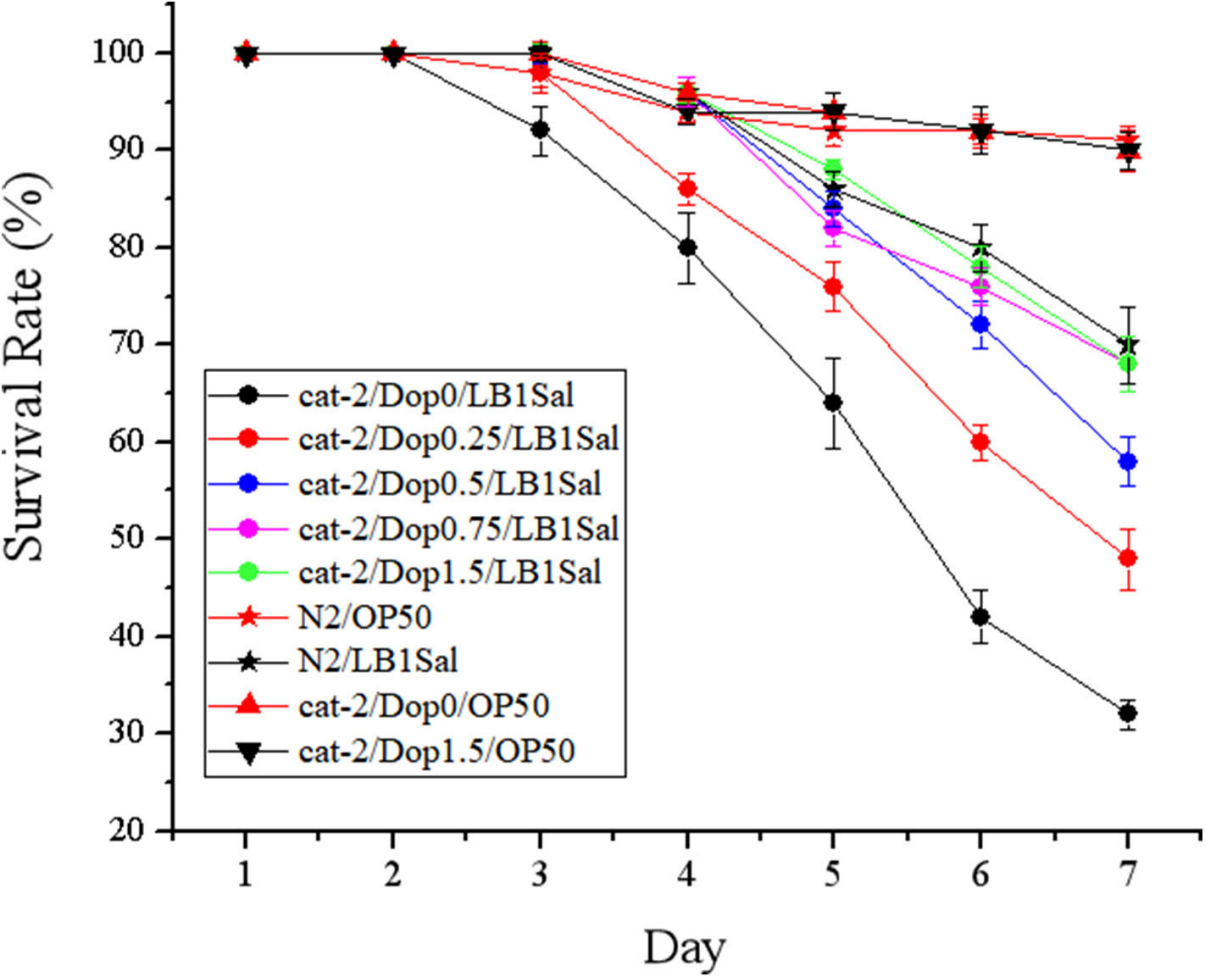
Dopamine resulted in the protection from *Lactobacillus zeae* LB1 to mutant *cat-2* against *Salmonella* Typhimurium DT104 infection. Synchronized L1 stage worms of mutant *cat-2* were treated with various concentration of dopamine (0, 0.25, 0.5, 0.75, and 1.5 mM) in nematode growth medium (NGM) agar seeded with OP50 until they reached L4 stage. And then the worms were treated with *L. zeae* LB1 for 18 h followed by DT104 infection for 7 days. Then life span was detected. N2: the wild type (WT); *cat-2*: mutant; Dop0: dopamine at 0 mM; Dop0.25: dopamine at 0.25 mM; Dop0.5: dopamine at 0.5 mM; Dop0.75: dopamine at 0.75 mM; Dop1.5: dopamine at 1.5 mM; LB1Sal: treated with *L. zeae* LB1 for 18 h followed by DT104 infection for 7 days; OP50: treated with *E. coli* OP50 only. Wild-type worms (N2) were used as control. The number of live worms in each group was recorded daily and calculated as follows: survival rate (%) = (live worms/total worms used) × 100. A worm was considered to be dead when it failed to respond to touch. Results are presented as mean ± S.D. with triplicate measurement.

The effects of supplementation of dopamine on restoring the protection of *L. zeae* LB1 are given in Figure 5. Again, *L. zeae* LB1 offered no protection to mutant *cat-2* when there was no supplementation of dopamine. However, the protection of *L. zeae* LB1 was generated by dopamine in a dose-dependent manner (0–1.5 mM). In particular, the survival rate (~70%) of mutant *cat-2* exposed to 0.75 and 1.5 mM of dopamine was comparable with that of the WT without exposure to dopamine when both were subjected to the pretreatment of *L. zeae* LB1 and DT104 infection. These results indicate that exogenous dopamine at 0.75 mM and above can fully restore the protection of *L. zeae* LB1 to the nematode mutant against *S*. Typhimurium DT104 infection.

To determine whether dopamine supplementation affected the viability of DT104 and *C. elegans* as well as the pathogenicity of DT104 in the life-span assay, the assays were performed with the WT nematode that had been either infected or not with DT104 in the absence and presence of dopamine (0.75 and 1.5 mM). At both the dopamine concentrations, *C. elegans* had a similar survival rate to the worms without dopamine treatment regardless if the nematode had been infected with DT104 or not. Similarly, the same level of growth of DT104 in a liquid medium was also observed regardless if dopamine (0.75 and 1.5 mM) was supplemented or not. These results indicate that exogenous dopamine at the tested concentrations had no effects on the viability of both DT 104 and *C. elegans* as well as the pathogenicity of DT104.

### Gene Expression of Mutant *cat-2* Responding to Dopamine Supplementation

**Figure 6:**
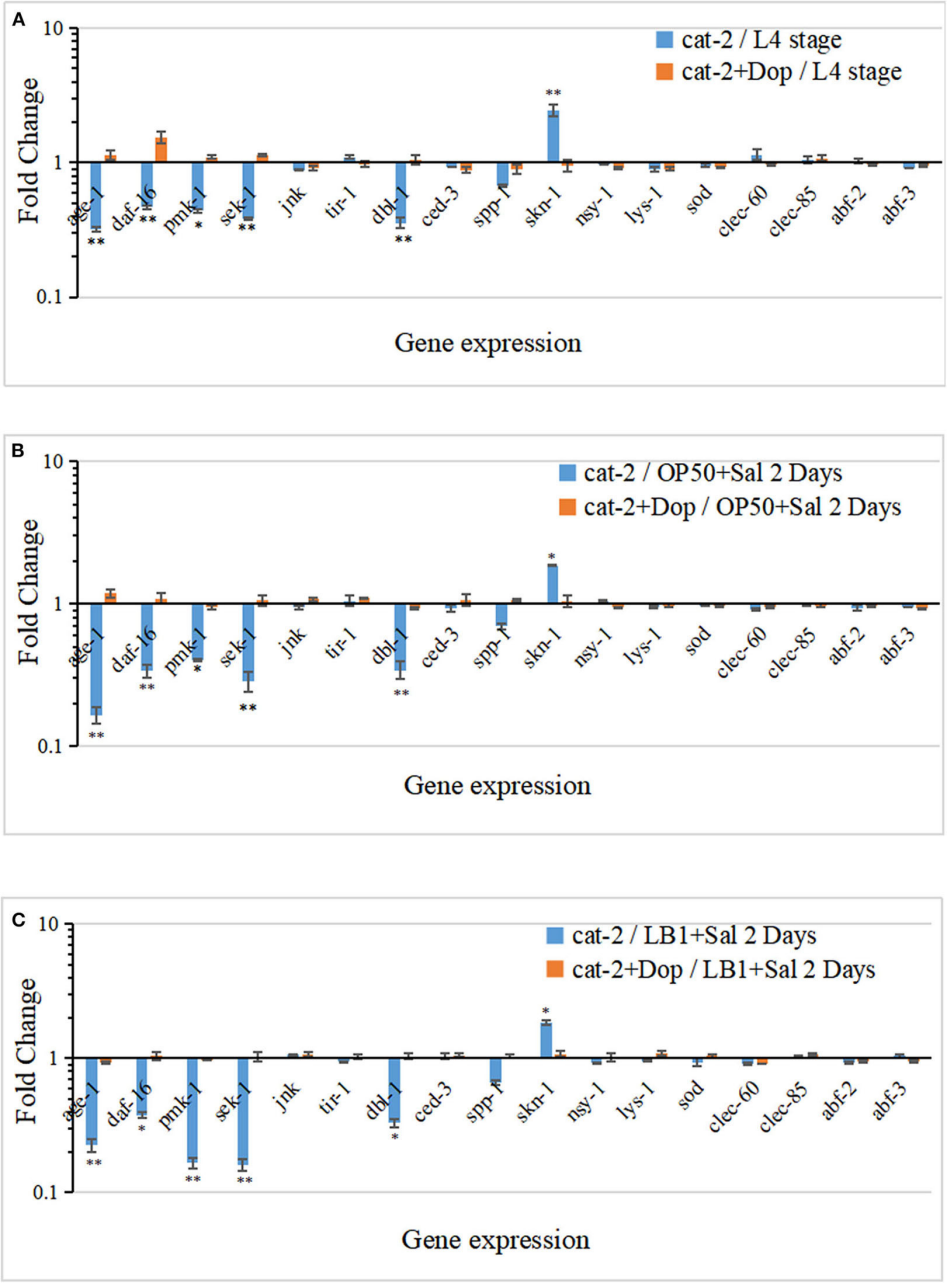
Gene expression response in dopamine-involved protection from *Lactobacillus zeae* LB1 to mutant *cat-2* against *Salmonella* Typhimurium DT104 infection. **(A)** Gene expression in L4 stage mutant *cat-2* treated with or without dopamine. cat-2/L4 stage: L4 stage *cat-2* worms; cat-2+Dop/L4 stage: L4 stage *cat-2* worms supplemented 1.5 mM of dopamine. **(B)** Gene expression in mutant *cat-2* infected with DT104 treated with or without dopamine. cat-2/OP50+Sal 2 Days: L4 stage *cat-2* worms were incubated with OP50 for 18 h and then infected with DT104 for 48 h; cat-2+Dop/OP50+Sal 2 Days: L4 stage *cat-2* worms were incubated with OP50 for 18 h and then infected with DT104 for 48 h, and 1.5 mM of dopamine was supplemented in the entire procedure. **(C)** Gene expression in mutant *cat-2* treated with or without dopamine that was also pretreated with *L. zeae* LB1. cat-2/LB1+Sal 2 Days: L4 stage *cat-2* worms were pretreated with *L. zeae* LB1 for 18 h and then infected with DT104 for 48 h; cat-2+Dop/LB1+Sal 2 Days: L4 stage *cat-2* worms were pretreated with *L. zeae* LB1 for 18 h and then infected with DT104 for 48 h, and 1.5 mM of dopamine was supplemented in the entire procedure. ΔCt represents the difference between the Ct value with the primers to a target gene and the Ct value to housekeeping genes. The ΔΔCt represents the difference between the ΔCt value of mutant *cat-2* group either in the presence or absence of dopamine and the ΔCt value of the wild type (WT) control group with the same treatment. The values derived from 2^−ΔΔCt^ represent fold changes of mutant *cat-2* groups in abundance relative to the WT group. The WT control groups had the 2^−ΔΔCt^ value of 1. Results are presented as mean ± S.D. with triplicate measurement. *P ≤ 0.05 for the same gene among different treatments, and **P ≤ 0.01.

To determine the cell signaling of mutant *cat-2* in response to the supplementation of dopamine in the life-span assay, the gene expression of major components in the p38-MAPK (*tir-1, nsy-1, sek-1*, and *pmk-1*) and IGF-1/DAF-16 (*age-1* and *daf-16*) pathways, previously identified antimicrobial peptides (*lys-7, spp-1, abf-2, clec-85, clec-60*, and *abf-3*), and other reported defense molecules (*sod-3, dbl-1*, and *skn-1*) was examined with the method described previously (Zhou et al., 2014, 2018). The experiment was carried out in three parallels. In parallel A, gene expression was examined in L4-stage *cat-2* worms supplemented without/with 1.5 mM of dopamine. As shown in Figure 6A, when incubated with *E. coli* OP50 alone, several genes in the IGF-1/DAF-16 (*age-*1 and *daf-16*) and p38-MAPK (*pmk-1* and *sek-1*) pathways or for defense functions (*dbl-1* and *spp-1*) were all down-regulated in mutant *cat-2* compared with the WT (*P* ≤ 0.05), and the supplementation of dopamine (1.5 mM) restored their expression back to the level in the WT (value = 1). One exception was the up-regulation of *skn-1* expression (involved in defense function) in the mutant, which substantially exceeded the expression level in the WT (*P* ≤ 0.05). However, the supplementation of dopamine (1.5 mM) down-regulated its expression back to the level observed in the WT.

In parallel B, L4-stage *cat-2* worms supplemented without/with 1.5 mM of dopamine were incubated with OP50 for 18 h followed by DT104 infection for 48 h, and the gene expression was then examined. Similarly, *age-1*, daf*-16, pmk-1, sek-1, dbl-1*, and *spp-1* all expressed at low levels in *S*. Typhimurium infected mutant *cat-2* (*P* ≤ 0.05), and the supplementation of dopamine (1.5 mM) also restored their expression back to the level in the WT (Figure 6B). Again, the expression of *skn-1* was up-regulated in the mutant in the absence of dopamine but down-regulated after dopamine supplement (*P* ≤ 0.05).

In parallel C, L4-stage *cat-2* worms supplemented without/with 1.5 mM of dopamine were treated with *L. zeae* LB1 for 18 h followed by DT104 infection for 48 h, and the gene expression was then examined. The six genes (*age-1, daf-16, pmk-1, sek-1, dbl-1*, and *skn-1*) demonstrated a similar pattern of gene expression profile (either down-regulation or up-regulation) in the mutant that had been subjected to both *S*. Typhimurium infection and *L. zeae* LB1 pretreatment before and after the supplementation of dopamine when compared with the WT (Figure 6C). It appears that the mutation in dopamine production induced the immune deficits and life-span shortening in *C. elegans*, and the supplementation of dopamine could attenuate the mutation effect.

## Discussion

Nematodes possess sensitive olfactory neurons sensing various odors and showing attraction, aversion, and memory of these odors (Bargmann et al., 1993). Aversive or attractive behavior may provide a window for protective responses to develop. Previous studies described that bacterial pathogens, such as *Serratia marcescens* ATCC 13880 and *Pseudomonas aeruginosa* PA14, can induce aversive olfactory behavior in *C. elegans* (Ha et al., 2010; Francesco et al., 2016; Jin et al., 2016). However, *S*. Typhimurium DT104 did not elicit aversive olfactory behavior of the nematode in the current study. Sharma et al. (2019) evaluated chemotactic behavior of *C. elegans* to 15 probiotic cultures using a binary choice index. Only four tested isolates were preferred by the nematode with 11 isolates less attractive compared with *E. coli* OP50. In the present study, similar observations were obtained with *L. zeae* LB1, which was much more attractive to *C. elegans* than both DT104 and *E. coli* OP50.

Olfactory learning and “memory” behaviors of *C. elegans* can be mediated by serotonergic circuits or dopamine signaling (Sawin et al., 2000; Anyanful et al., 2009). While mutation in *tph-1* is deficient in tryptophan hydroxylase that is required for biosynthesis of serotonin (Sze et al., 2000), *cat-2* encodes tyrosine hydroxylase, the rate-limiting enzyme in dopamine biosynthesis (Lints and Emmons, 1999). The use of both *cat-2* mutant and *tph-1* mutant was to distinguish their roles and possible mechanisms (Anyanful et al., 2009). In the present study, two mutants defective in either *tph-1* or *cat-2* exhibited similar choosing behavior to the WT of *C. elegans*, suggesting that nematode preference to *L. zeae* LB1 did not involve serotonin- or dopamine-mediated motivation or memory behavior.

*Bacillus licheniformis* was reported to enhance the resistance of *C. elegans* to the infection of *Staphylococcus aureus* (Yun et al., 2014), and the effect on the longevity of *C. elegans* was via host serotonin signaling (Park et al., 2015). In the present study, both *tph-1* and *cat-2* mutants succumbed faster than the WT to *S*. Typhimurium DT104 infection, suggesting a positive role of both serotonin and dopamine in the nematode defense against the pathogen infection. Furthermore, it has been observed that the resistance of mutant *cat-*2 to *S*. Typhimurium infection was increased by supplementation of dopamine, but not by pretreatment with *L. zeae* LB1, and that the protection to mutant *cat-*2 from *L. zeae* LB1 was also restored by dopamine. These data suggested that dopamine plays an essential role in the resistance of *C. elegans* to *S*. Typhimurium infection, including the protection from *L. zeae* LB1.

Cao and Aballay (2016) reported that dopamine signaling in *C. elegans* negatively regulated the nematode innate immune response to *P. aeruginosa* infection through a D1-like dopamine receptor, DOP-4, by down-regulating the p38/PMK-1 MAPK pathway. When the nematode was treated with the dopamine antagonist chlorpromazine, it became more resistant to *P. aeruginosa* infection. However, the resistance was significantly reduced by exogenous dopamine. A negative regulation of innate immune response in *C. elegans* by serotonin was also described by Anderson et al. (2013), in which serotonin signaling limited the rate of pathogen clearance of *Microbacterium nematophilum* through regulation of G-protein signaling. In the present study, both *cat-2* and *tph-1* mutants succumbed faster than the WT to *S*. Typhimurium DT104 infection. In addition, exogenous dopamine was able to enhance the resistance of mutant *cat-2*, although the effect of exogenous serotonin on the resistance of mutant *tph-1* was not examined. These data imply that both dopamine and serotonin positively regulate the nematode innate immune response to *S*. Typhimurium DT104 infection, which is obviously different from the observations reported by Cao and Aballay (2016) and by Anderson et al. (2013). In agreement with our findings, a positive regulation of innate immune response by serotonin signaling in *C. elegans* has been documented in response to the infection of *P. aeruginosa* PA14 (Zhang et al., 2005; Gravato-Nobre and Hodgkin, 2010). Worms that lack *tph-1* were more susceptible to PA14 than WT animals. It is unknown at present what the potential causes are for the different findings. One possibility could be attributed to the different host responses of *C. elegans* to different bacterial pathogens or even to different isolates within the same species. To clarify the issue, further in-depth and well-controlled comparison studies are required.

It has been shown that neurotransmitters can regulate immune responses. A regulatory axis from the gut microbes to neurotransmitter and then to autoimmune system was proposed in both rodents and humans (Tognini, 2017). Xue et al. (2018) showed that depletion of dopaminergic neurons significantly promoted activation of mice hepatic iNKT cells and augmented concanavalin A-induced liver injury by D1-like receptor-PKA pathway in mice. In the present study, expression of the genes in IGF-1/DAF-16 signaling (*age-1* and *daf-16*) and innate immune signaling pathways (*pmk-1* and *sek-1*) was significantly down-regulated in the mutant defective in *cat-2*, and supplementation of dopamine to mutant *cat-2* restored the expression of these genes to the level in the WT. These results appear to support the notion that dopaminergic signaling has a role in regulating the IGF-1/DAF-16 and innate immune signaling pathways in *C. elegans*.

The degree of host susceptibility of *C. elegans* to various pathogens or toxins was based on the expression level of genes with a defense function, which was mediated by the IGF/DAF-16 or p38-MAP kinase signaling pathways (Troemel et al., 2006). In the present study, the shorter life span and down-regulation of defense-related genes in the *S*. Typhimurium infected mutant worms (*cat-2*) suggest a role of the IGF-1/DAF-16 and innate immune signaling pathways mediated by dopamine in the nematode resistance, which is consistent with the report that *C. elegans* may use a neuronal circuit to regulate protective functions via longevity and innate immune signaling pathways to combat bacterial infection when encountered with bacterial pathogens. Similarly, Anyanful et al. (2009) found that EPEC toxins or other pathogens triggered a neuronal circuit mediated by dopaminergic neurons that activated DAF-16 and PMK-1 signaling, which in turn activated other genes to protect *C. elegans*. In addition, Kamaladevi and Balamurugan (2016) reported that *Lactobacillus casei* triggered a TLR-mediated p38 MAPK pathway to increase host resistance and protect nematodes against pathogen infection. In view of these descriptions, our data support the same notion; i.e., the nervous and immune systems can communicate and influence each other in order for the nematode to rapidly and effectively protect themselves from bacterial infection. Given that the signaling pathways are highly conserved from *C. elegans* to humans (Weidhaas et al., 2006), the functions of neurotransmitters reported herein in regulating cell signaling and defense of the nematode may serve as a reference for developing bacterial control strategies in mammalians.

In summary, the present study has demonstrated that both serotonin and dopamine play a positive role in the host defense of *C. elegans* to *S*. Typhimurium infection. The protection offered by *L. zeae* LB1 was not dependent on modifying olfactory preference of the nematode but was mediated by dopamine. In addition, both p38-MAPK and IGF-1/DAF-16 signaling pathways appeared to be involved in the regulation of the protection.

## Data Availability Statement

The raw data supporting the conclusions of this article will be made available by the authors, without undue reservation.

## Author Contributions

XL, LJ, HY, and JG designed the experiments. XL, LJ, and LL performed the research. XL, LJ, LL, HY, and JG analyzed data. SN and MX contributed to discussion and resource provision. XL, LJ, and JG wrote the manuscript. JG and MX conceived the research. All the authors approved the final version of manuscript.

## Conflict of Interest

The authors declare that the research was conducted in the absence of any commercial or financial relationships that could be construed as a potential conflict of interest.
